# Isolation, Diversity, and Antimicrobial and Immunomodulatory Activities of Endophytic Actinobacteria From Tea Cultivars Zijuan and Yunkang-10 (*Camellia sinensis* var. *assamica*)

**DOI:** 10.3389/fmicb.2018.01304

**Published:** 2018-06-18

**Authors:** Wei Wei, Yu Zhou, Fanjie Chen, Xiaomei Yan, Yongmin Lai, Chaoling Wei, Xiaoyun Chen, Junfeng Xu, Xu Wang

**Affiliations:** ^1^State Key Laboratory Breeding Base for Zhejiang Sustainable Pest and Disease Control, Institute of Quality and Standard for Agro-Products, Zhejiang Academy of Agricultural Sciences, Hangzhou, China; ^2^State Key Laboratory of Tea Plant Biology and Utilization, Anhui Agricultural University, Hefei, China

**Keywords:** *Camellia sinensis*, endophytic actinobacteria, diversity, antimicrobial activity, immunomodulatory activities

## Abstract

Endophytic actinobacteria exist widely in plant tissues and are considered as a potential bioresource library of natural products. Tea plants play important roles in human health and in the lifestyles of Asians, especially the Chinese. However, little is known about the endophytic actinobacteria of tea plants. In this study, 16 actinobacteria of 7 different genera and 28 actinobacteria of 8 genera were isolated and analyzed by 16S rRNA gene sequencing from tea cultivars of Zijuan and Yunkang-10 (*Camellia sinensis* var. *assamica*), respectively. The diversity of actinobacteria species from Zijuan were higher in July than December (6 vs. 3 genera), but the diversity of species from Yunkang-10 were higher in December than July (7 vs. 3 genera). No actinobacteria isolates were obtained from any tea cultivar in September. Ten isolates from Yunkang-10 exhibited antimicrobial activity against at least one human pathogenic microorganism (*Staphylococcus epidermidis*, *Shigella flexneri*, and *Escherichia coli*), but none of the isolates from Zijuan exhibited antimicrobial activities. Fourteen strains were further exammined the genes of polyketide synthetase (*PKS*)*-I* and *PKS-II* and non-ribosomal peptide synthetase (*NRPS*). *Brevibacterium* sp. YXT131 from Yunkang-10 showed strong inhibitory activity against *S. epidermidis*, *Sh. flexneri*, and *E. coli*, and *PKS-I* and *PKS-II* and *NRPS* genes were obtained from the strain. In *in vitro* assays, extracts from 14 actinobacteria that were tested for antibiotic biosynthetic genes showed no inhibition of concanavalin A (ConA)-induced murine splenocyte proliferation. In *in vivo* assays, the crude extract of YXT131 modulated the immune response by decreasing the proinflammatory cytokines interleukin (IL)-12/IL-23 p40 and tumor necrosis factor (TNF)-α in the serum of mice. These results confirm that endophytic actinobacteria from tea plants might be an undeveloped bioresource library for active compounds.

## Introduction

Actinobacteria are aerobic, gram-positive bacteria and are well-known producers of a vast array of secondary metabolites, including antibiotics, immunosuppressive agents, antitumor agents, and enzymes, many of which are of great importance to the pharmaceutical and agricultural industries (Saini et al., 2015; Landwehr et al., 2016; Salcedo et al., 2016; Yang et al., 2016). Endophytes are microorganisms that ubiquitously colonize the internal tissues of plants without causing any negative effects, and some endophytes are able to control plant pathogens and promote the growth of plants (Santoyo et al., 2016; Kandel et al., 2017). Although numerous species of actinobacteria occur in the soil, other microbial habitats, such as leaf litter and plants, are potential sources of actinobacteria for the isolation of biologically active compounds (Sardi et al., 1992; Takahashi and Omura, 2003; Gos et al., 2017). In recent years, endophytic actinobacteria have been isolated from many crop plants (such as wheat, rice, and potatoes) (Coombs and Franco, 2003; Sessitsch et al., 2004; Tian et al., 2007) and medicinal plants (Qin et al., 2009; Gos et al., 2017). Endophytic actinobacteria are a potential source for the production of secondary metabolites that are used in the direct antagonism of pests and diseases (Cao et al., 2005) as well as various natural products with antimicrobial, antitumor, and anti-infection activities (Qin et al., 2011; Gos et al., 2017). Endophytic actinobacteria can also confer salt tolerance to host plants and promote host-plant growth (Qin et al., 2017).

As a popular non-alcoholic beverage, tea and tea drinks play important roles in human health and lifestyle, such as by reducing cardiovascular mortality and treating digestive disorders (Yang C.S. et al., 2009; Persson et al., 2010; Begas et al., 2017). As the same plant species, the tea cultivars of Zijuan and Yunkang-10 belong to the taxonomic species of *Camellia sinensis* var. *assamica*, which originated from the Yunnan province of China (Wang et al., 2017; Zhu et al., 2017). The two closely related cultivars of Zijuan and Yunkang-10 are the major raw materials for Pu’er tea (a kind of dark tea) produced in Yunnan. Catechins are the major secondary metabolic products in tea leaves (especially in green tea), and polyphenols are known to confer health benefits (Khan and Mukhtar, 2007). Anthocyanin is another functional flavonoid with health benefits and is widely distributed in higher plants (e.g., vegetables, flowers, fruits, cereals, and tea plants) (Joshi et al., 2015; Fabroni et al., 2016; Chen et al., 2017). The total anthocyanin content in Zijuan has been reported to be almost three times that found in other Chinese tea cultivars (including Yunkang-10), and thus Zijuan is considered to be an anthocyanin-rich cultivar (Yang X.R. et al., 2009). However, only a few studies have observed on tea plant endophytes, and those studies have mainly been performed on endophytic fungi and for plant disease protection (Rabha et al., 2014). For example, the endophytic fungus *Colletotrichum gloeosporioides* from healthy tea plant leaves shows strong inhibitory activity against tea pathogens of *Pestalotiopsis theae* and *Colletotrichum camelliae*; the inhibitory factors may be the highly efficient fungal chitinase and protease (Rabha et al., 2014). Compared to endophytic fungi, endophytic actinobacteria have received almost no attention in tea plants, and the interactions between endophytic actinobacteria and tea plants have not been investigated. Based on recent research progress in other plants, the study of endophytic actinobacteria culture and bioactivity is of great significance for developing bioresource libraries. In this study, the endophytic actinobacteria from tea plants of Zijuan and Yunkang-10 were isolated at different seasons, and the bioactivities of antimicrobial and immunomodulatory were screened and further evaluated for the isolates.

## Materials and Methods

### Tea Sample Collection

Three-year-old tea plants of Zijuan and Yunkang-10 (*Camellia sinensis* var. *assamica*) were obtained from Dechang tea plantation (Shucheng China, 31°11’ N, 116°47’ E). The leaf samples of Zijuan and Yunkang-10 were collected on July 5, September 10, and December 5 of 2015. At the each time of sampling, 15 branches with the same growth and no pest damage were selected for each tea cultivar (considered as 1 sample). The healthy branches were placed in fully soaked floral foam and transported to the laboratory within 2 h for actinobacteria isolation; the isolation procedures were performed within 96 h.

### Endophytic Actinobacteria Isolation

The leaf samples were pretreated following the method described by Qin et al. (2009) with minor modifications. The samples were air-dried for 48 h at room temperature and then washed with ultrasonic cleaning (160 W, 15 min). After drying, the samples were sterilized in the following order: a 6-min wash in 5% NaOCl, followed by a 10-min wash in 2.5% Na_2_S_2_O_3_, a 5-min wash in 70% ethanol, a 5-min wash in sterile water, and a final rinse in 10% NaHCO_3_ for 10 min. The sterilized tissues were imprinted on nutrient agar (NA, Difco) and tryptic soy agar (TSA, Difco), and incubated at 28°C for 2 weeks to ensure the sterilization effectiveness. After surface sterilization and thorough drying under aseptic conditions, the samples were cut up in a sterile mortar and ground to a homogenate, followed by dilution to 10^-1^ to 10^-3^ with sterile water. Aliquots of 200 μL of the dilutions were spread-plated onto a series of isolation media as indicated in **Table 1** and incubated at 28°C for 2–3 weeks for actinobacteria cultivation. The pH of the selected media was adjusted to 7.2. Each isolation medium was amended with nalidixic acid (50 mg/L) and nystatin (100 mg/L) to prevent the growth of gram-negative bacteria and fungi. As colonies appeared on the plates, candidate colonies were observed and selected carefully according to phenotypic characteristics.

**Table 1:** Culture medium composition for endophytic actinobacteria isolation.

Medium	Composition (per 1000 mL)	Reference
GAUZE’s medium	5 g K_2_HPO_4_, 20 g soluble starch, 0.5 g MgSO_4_⋅7H_2_O, 0.01 g FeSO_4_⋅7H_2_O, 1 g KNO_3_, 0.5 g NaCl, 18 g agar	Ma et al., 2017
TWYE	0.5 g K_2_HPO_4_, 0.25 g yeast extract, 18 g agar	Crawford et al., 1993
YECD	2 g K_2_HPO_4_, 0.3 g yeast extract, 0.3 g glucose, 18 g agar	Coombs and Franco, 2003
Humic acid-vitamin agar (HV)	0.02 g CaCO_3_, 0.5 g Na_2_HPO_4_, 0.5 g MgSO_4_⋅7H_2_O, 0.01 g FeSO_4_⋅7H_2_O, 1 g Humic acid, 1.7 g KCl, 0.5 mg VB_6_, 0.5 mg p-aminobenzoic acid, 0.5 mg riboflavin, 0.5 mg thiamine, 0.5 mg inositol, 0.5 mg pantothenic acid, 0.5 mg nicotinic acid, 0.25 mg biotin, 18 g agar	Hayakawa and Nonomura, 1989
Glucose-Asparagine modified media (GA)	1 g K_2_HPO_4_, 1 g asparagine, 0.01 g ZnSO_4_⋅7H_2_O, 0.01 g FeSO_4_⋅7H_2_O, 10 g glucose, 0.01 g MnCl_2_⋅4H_2_O, 18 g agar	Shirling and Gottlieb, 1966


### DNA Extraction, 16S rRNA Gene Sequencing, and Phylogenetic Analysis

The obtained isolates were subjected to 16S rRNA gene sequence analysis for genus and species identification. The genomic DNA was extracted using the method of Li et al. (2007). The 16S rRNA gene of each isolate was amplified using primer pairs 27F and 1492R (**Table 2**), and polymerase chain reaction (PCR) amplification was carried out as described by Li et al. (2007). The reagents for PCR reaction were purchased from TaKaRa (Dalian, China). The PCR -products were separated by agarose gel electrophoresis, purified using QIA quick gel extraction kits (Qiagen, Hilden, Germany), then ligated into a pMD-19T vector (TaKaRa). Positive clones were screened further, and insert DNA sequencing was performed by Invitrogen (Shanghai, China) on an Applied Biosystems PRISM 3730 DNA sequencer.

**Table 2:** Polymerase chain reaction (PCR) primers used in this study.

Primer name	Sequence (5′—3′)	Target gene	Length (bp)	Reference
27F	5′-AGAGTTTGATCCTGGCTCAG-3′	*16S rRNA*	1400–1500	Li et al., 2007
1492R	5′-ACGGTTACCTTGTTACGACTT-3′			
K1F	5′-TSAAGTCSAACATCGGBCA-3′	*PKS-I*	1200–1400	Ayuso-Sacido and Genilloud, 2005
M6R	5′-CGCAGGTTSCSGTACCAGTA-3′			
KS_α_	5′-TSGCSTGCTTGGAYGCSATC-3′	*PKS-II*	600	Metsa-Ketela et al., 1999
KS_β_	5′-TGGAANCCGCCGAABCCTCT-3′			
A3F	5′-GCSTACSYSATSTACACSTCSGG-3′	*NRPS*	700–800	Ayuso-Sacido and Genilloud, 2005
A7R	5′-SASGTCVCCSGTSCGGTAS-3′			


The 16S rRNA gene analysis was performed by BLAST searches in the National Center for Biotechnology Information database^1^ and EzBioCloud^2^. Multiple sequence alignment of selected 16S rDNA sequence was carried out using CLUSTAL_X (version 2.0) (Thompson et al., 1997), and a phylogenetic tree was constructed using MEGA v6.0 (Tamura et al., 2013). Distances (distance options according to the Kimura two-parameter model) (Kimura, 1980) and clustering were based on the neighbor-joining (Saitou and Nei, 1987) method. Bootstrap analysis based on 1000 resamplings was used to evaluate the topology of the neighbor-joining tree (Felsenstein, 1985). The 16S rRNA gene sequences of the 44 isolates have been deposited in GenBank under the accession numbers (MH298662–MH298705).

### Detection of *PKS-I*, *PKS-II*, and *NRPS* Genes

Three sets of degenerate primers for amplification of the genes encoding polyketide synthases I and II (*PKS-I* and *PKS-II*) and non-ribosomal peptide synthetase (*NRPS*) were selected (**Table 2**), and amplification was carried out as recommended by Metsa-Ketela et al. (1999) and Ayuso-Sacido and Genilloud (2005). The reaction mixture contained 2.5 U of *Taq* DNA polymerase, 1 mM MgCl_2_, 0.4 mM deoxynucleoside triphosphates, 2 μM each primer, and 5% dimethyl sulfoxide in a 50-μL reaction volume. A reaction mixture with no actinobacterial DNA template was used as a negative control. Thermocycling conditions consisted of one denaturation step of 94°C for 5 min, 30 amplification cycles of 94°C for 1 min, 57°C (for K1F–M6R and A3F–A7R) or 58°C (for KS_α_–KS_β_) for 1 min, and 72°C for 2 min; and a final extension at 72°C for 5 min.

### Active Compound Extraction and Bioactivity Evaluation

The endophytic isolates were cultured in GAUZE’s liquid medium at 28°C and 180 r/min (Rehacek, 1959). After 7–12 days of cultivation, the 100 mL culture broth was collected by centrifugation at 12,000 ×*g* for 10 min and extracted by 100 mL ethyl acetate for three times. The organic phase was evaporated under reduced pressure to yield a dry extract. The dry extract was resuspended by 5 mL sterile water and used for antimicrobial screening. The antimicrobial susceptibility was examined by placing antimicrobial testing disks (7 mm diameter) containing 25 μL test extract suspension onto LB plates (Mearns-Spragg et al., 1998). The tested plates were incubated at 37°C, and the diameters of the inhibition zones were measured after 24 h. A 25-μL volume of sterile water was used as a negative control. The pathogenic bacteria *Staphylococcus epidermidis*, *Shigella flexneri*, *Escherichia coli*, and *Bacillus cereus* were used as the indicator microorganisms for antimicrobial determination. The pathogenic microorganisms were obtained from the Institute of Quality and Standard for Agro-products, Zhejiang Academy of Agricultural Sciences.

### Animal Experiments and Physiological Tests

Female BALB/c mice at 8–9 weeks of age were purchased from the Zhejiang Laboratory Animal Center (Hangzhou, China). The mice were maintained in pathogen-free conditions with standard laboratory chow and water *ad libitum*. Animal experiments were approved and performed in accordance with the guidelines of the Animal Care Committee of Zhejiang province, China. Dried extracts from the test endophytic actinobacteria were resuspended by saline with concentration of 2 mg/mL and injected i.p. 100 μL per mouse once daily for 2 weeks. The same volume (100 μL per mouse) of saline was given as a vehicle control. Three independent experiments were repeated, and in each experiment five mice were used. Clinical signs of poisoning were assessed and the weights of mice were recorded daily. Blood samples (∼500 μL per mouse) were taken from the retro-orbital venous plexus at the end of the experiment and incubated at 4°C for 30 min. Serum was collected by centrifugation at 4500 ×*g* for 10 min and stored at -20°C until analysis. Cytokines [interleukin-2 (IL-2), interleukin-6 (IL-6), the shared p40 subunit of IL-12 and IL-23 (IL-12/IL-23 p40), and tumor necrosis factor-alpha (TNF-α)] in the serum were determined with sandwich enzyme-linked immunosorbent assay (ELISA) kits according to the manufacturer’s instructions (Dakewe Biotech, Shenzhen, China).

### Splenocyte Proliferation

Freshly isolated splenocytes were obtained from BALB/c mice and incubated in 200 μL RPMI 1640 with 10% FBS, 100 U/mL penicillin, 100 μg/mL streptomycin, and 5 μg/mL of concanavalin A (ConA) in a humidified, 37°C, 5% CO_2_-containing incubator for 48 h in the presence or absence of extracts. Cyclosporin A (CsA) (500 ng/mL) was used as a positive control. Three independent experiments were repeated, and in each experiment five wells of splenocytes were used. RPMI 1640 medium was purchased from Gibco, Thermo Fisher Scientific (Waltham, MA, United States). ConA and CsA were purchased from Sigma (St. Louis, MO, United States). The cell number was determined by a Millipore Guava easyCyte 8HT Flow Cytometer (Millipore, Billerica, MA, United States).

### Statistical Analysis

Data are presented as means ± standard deviation. Statistical analyzes were performed using Student’s *t* test. *P* < 0.05 was considered statistically significant.

### Ethics Statement

This study was carried out in accordance with the guidelines of the Animal Care Committee of Zhejiang province, China (Government Decree No. 263). The protocol was approved by the Committee on the Ethics of Animal Experiments of Zhejiang Academy of Agricultural Science.

## Results

### Evaluation of Surface Sterilization

Surface sterilization is critical for the study of plant endophytic actinobacteria. In this study, the surface-sterilized leaves were examined by NA and TSA, and no microbial colony was observed after 2 weeks of incubation at 28°C. This indicated that the surface-sterilization protocol modified from Qin et al. (2009) was effective in removing phyllospheric microorganisms of tea plants.

### Selective Isolation of Culturable Endophytic Actinobacteria From Zijuan and Yunkang-10

To obtain as many endophytic actinobacteria as possible, five selective isolation media were used simultaneously in this study (**Table 1**). Endophytic actinobacteria were isolated on all of five media. In total, 44 actinobacterial strains (28 from Yunkang-10 and 16 from Zijuan) were isolated from 3 samples of Zijuan and 3 samples of Yunkang-10 (**Table 3**).

**Table 3:** The isolated endophytic actinobacteria of Zijuan and Yunkang-10.

Tea cultivars	July		September		December	
	Species	Number	Species	Number	Species	Number
Zijuan	*Brachybacterium* sp.	1			*Brachybacterium* sp.	2
	*Brevibacterium* sp.	1			*Kocuria* sp.	3
	*Kocuria* sp.	1			*Microbacterium* sp.	1
	*Leucobacter* sp.	1				
	*Micrococcus* sp.	4				
	*Streptomyces* sp.	2				
	Total number	10	Total number	0	Total number	6
Yunkang-10	*Brevibacterium* sp.	4			*Pseudarthrobacter* sp.	1
	*Micrococcus* sp.	1			*Brachybacterium* sp.	1
	*Mycobacterium* sp.	3			*Brevibacterium* sp.	6
					*Kocuria* sp.	4
					*Microbacterium* sp.	5
					*Micrococcus* sp.	2
					*Saccharomonospora* sp.	1
	Total number	8	Total number	0	Total number	20


### Diversity of Endophytic Actinobacteria Analyzed by 16S rRNA Gene Sequencing

**FIGURE 1:**
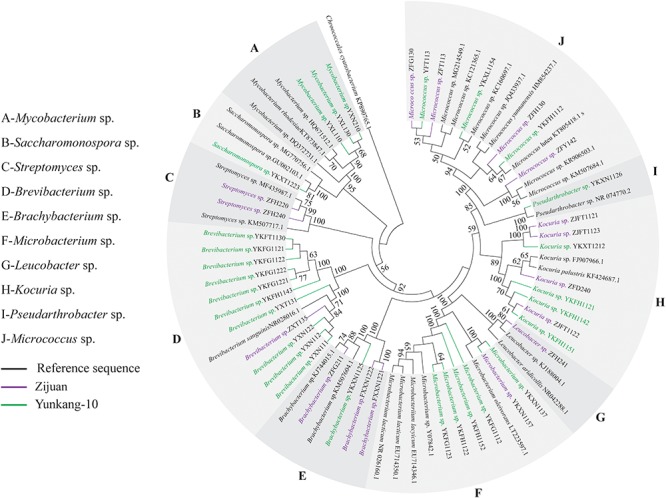
Phylogenetic analysis of endophytic actinobacteria in different seasons from Zijuan and Yunkang-10. The phylogenetic tree was constructed by using 73 16S rRNA gene sequences.

The endophytic actinobacteria obtained from Zijuan were distributed among 7 genera [i.e., *Brachybacterium* sp. (3 isolates), *Brevibacterium* sp. (1 isolate), *Kocuria* sp. (4 isolates), *Leucobacter* sp. (1 isolate), *Micrococcus* sp. (4 isolates), *Microbacterium* sp. (1 isolate), and *Streptomyces* sp. (2 isolates)] within the class actinobacteria (**Figure 1**). Among them, *Brachybacterium* sp. and *Kocuria* sp. were two mutual groups both isolated in July and December, while the others were only isolated in July or December, and no endophytic actinobacteria were obtained in September. The diversity of endophytic actinobacteria from the Zijuan cultivar was in the order of July (6 genera) > December (3 genera) > September (0 genera). The 28 endophytic actinobacteria isolated from Yunkan-10 were distributed among 8 genera [i.e., *Brevibacterium* sp. (10 isolates), *Micrococcus* sp. (3 isolates), *Mycobacterium* sp. (3 isolates), *Pseudarthrobacter* sp. (1 isolate), *Brachybacterium* sp. (1 isolate), *Kocuria* sp. (4 isolates), *Microbacterium* sp. (5 isolates), and *Saccharomonospora* sp. (1 isolate)] within the class actinobacteria (**Figure 1**). Isolates of *Brevibacterium* sp. and *Micrococcus* sp. were obtained from both July and December specimens, and isolates of *Mycobacterium* sp. were found only in July. The isolates from other genera were only obtained in December, while no endophytic actinobacteria were obtained in September. The diversity of endophytic actinobacteria from Yunkang-10 was in the order of December (7 genera) > July (3 genera) > September (0 genera). In comparing the two cultivars, isolates of *Leucobacter* sp. and *Streptomyces* sp. were endemic actinobacterial groups to the Zijuan cultivar, while *Mycobacterium* sp., *Pseudarthrobacter* sp., and *Saccharomonospora* sp. were endemic to Yunkang-10.

### Endophyte Antimicrobial Activity and Sequencing of *PKS* and *NRPS* Genes

**FIGURE 2:**
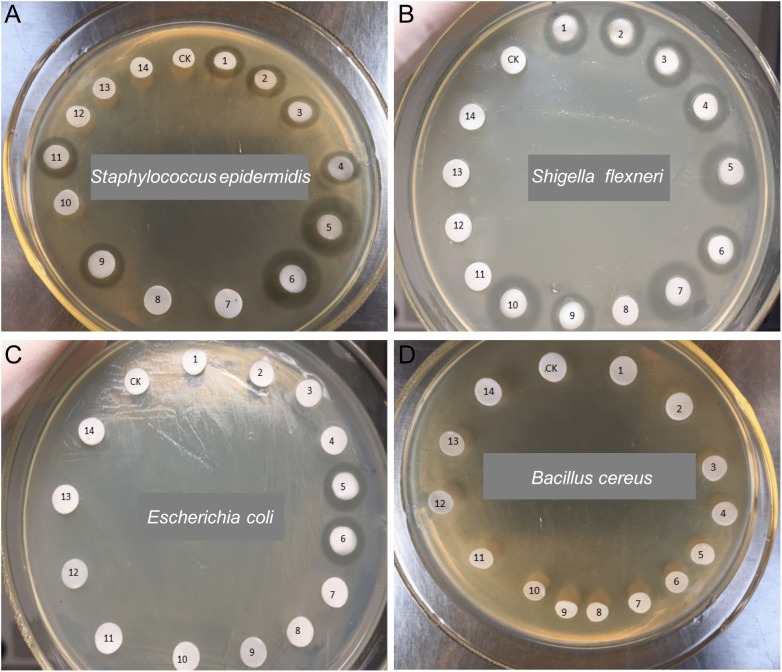
Antimicrobial activities of endophytic actinobacteria against pathogenic bacteria. *Staphylococcus epidermidis* **(A)**, *Shigella flexneri* **(B)**, *Escherichia coli* **(C)**, and *Bacillus cereus* **(D)** were the tested strains. CK-Vehicle, 1-YFT113, 2-YXN120, 3-YXN111, 4-YXN112, 5-YXT131, 6-YKFG1221, 7-YKFG1121, 8-YKFG1122, 9-YKFT1130, 10-YKFH1122, 11-YKFG1112, 12-ZFY142, 13-ZFG130, 14-ZJFT1121.

In antimicrobial screening test, several culture media indicated in **Table 1** were preliminary evaluated, and GAUZE’s medium was the best one for the majority of actinobacteria isolates from Zijuan and Yunkang-10. The extracts from GAUZE’s medium showed no obvious antimicrobial difference from other media, but all the tested strains could be cultured by the GAUZE’s medium. The 44 isolates were screened for antimicrobial activities against the pathogenic bacteria *S. epidermidis*, *Sh. flexneri*, *E. coli*, and *B. cereus.* Ten of the 28 isolates (35.7%) from Yunkang-10 exhibited activity against at least one of the tested pathogenic microorganisms. Surprising, none of the 16 isolates from Zijuan showed obvious antimicrobial activity. A total of 14 isolates selected (10 antimicrobial positive strains and four negative strains) were selected for the determination of antibiotic biosynthetic gene sequences of *PKS-I*, *PKS-II*, and *NRPS* by PCR amplification using specific primer sets K1F–M6R, KS_α_–KS_β_, and A3F–A7R, respectively (**Table 4**). As shown by primary screening, the inhibitory effect on *Sh. flexneri* was the most frequent detected antimicrobial activity in this study. Eight isolates were active against *S. epidermidis*, and 7 isolates were found to inhibit two or more pathogenic microorganisms. However, none of the isolates of this study exhibited activity against *B. cereus.* Two isolates, YXT131 and YKFG1221, which belong to the genus *Brevibacterium*, appeared to have a broad spectrum of antimicrobial activity (three pathogenic microorganisms). *Brevibacterium* sp. YXT131 exhibited high inhibitory effects against *S. epidermidis*, *Sh. flexneri*, and *E. coli* (**Figure 2** and **Table 5**).

**Table 4:** Endophytic actinobacteria isolated from Zijuan and Yunkang-10, and similarity values for 16S rRNA gene sequences.

Isolate no.	Sequence length	Closest cultivated species	Similarity (%)	Query coverage (%)
YFT113	1345	*Micrococcus yunnanensis* (FJ214355)	99.33	93.0
YXN120	1334	*Brevibacterium bullata* (D12785)	99.77	96.5
YXN111	1441	*Brevibacterium celere* (AY228463)	99.44	97.8
YXN112	1441	*Brevibacterium celere* (AY228463)	99.44	97.9
YXT131	1395	*Brevibacterium celere* (AY228463)	99.78	96.1
YKFG1221	1531	*Brevibacterium casei* (X76564)	99.72	100
YKFG1121	1489	*Brevibacterium casei* (X76564)	99.24	100
YKFG1122	1489	*Brevibacterium casei* (X76564)	99.31	100
YKFT1130	1492	*Brevibacterium casei* (X76564)	99.72	100
YKFH1122	1487	*Microbacterium lacticum* (X77441)	99.73	100
YKFG1112	1487	*Microbacterium lacticum* (X77441)	99.59	100
ZFY142	1428	*Micrococcus endophyticus* (EU005372)	97.88	99.0
ZFG130	1366	*Micrococcus yunnanensis* (FJ214355)	99.19	94.5
ZJFT1121	1459	*Kocuria marina* (AY211385)	99.86	100


**Table 5:** Antimicrobial activities and *PKS*/*NRPS* genes of culturable actinobacteria from Zijuan and Yunkang-10.

Isolate no.	Activity^a^ against				Presence^b^ of gene		
	*S. epidermidis*	*Sh. flexneri*	*E. coli*	*B. cereus*	*PKS-I*	*PKS-II*	*NRPS*
YFT113	++	++	-	-	+	-	-
YXN120	++	++	-	-	+	-	-
YXN111	++	++	-	-	+	-	-
YXN112	+	++	-	-	+	-	-
YXT131	+++	+++	++	-	+	+	+
YKFG1221	+++	+	++	-	+	+	+
YKFG1121	-	+++	-	-	+	+	-
YKFG1122	-	-	-	-	-	-	-
YKFT1130	+	+	-	-	+	+	-
YKFH1122	-	++	-	-	-	-	+
YKFG1112	+	-	-	-	-	-	+
ZFY142	-	-	-	-	-	+	-
ZFG130	-	-	-	-	+	-	-
ZJFT1121	-	-	-	-	-	+	-


*^*a*^Estimated by measuring the diameter of the clear zone of growth inhibition. Symbols: -, no activity; +, ++, and +++, weak activity, moderate activity, and strong activity, respectively. ^*b*^+, present; -, absent.*

The *PKS-I* sequence was detected in 9 isolates (64.3%), while the *PKS-II* and *NRPS* sequences were detected in 6 and 4 of the 14 strains (**Table 5** and **Supplementary Figure S1**), respectively. The isolates YXT131 and YKFG1221, which have broad spectrum antimicrobial activity, gave positive amplification products with *PKS-I*, *PKS-II*, and *NRPS* primers. The isolates from Yunkang-10 that exhibited antimicrobial activity against pathogenic microorganisms also gave positive amplification products for at least one of the *PKS-I*, *PKS-II*, and *NRPS* genes. The isolate YKFG1122 neither exhibited antimicrobial activity to the four tested pathogenic microorganisms nor provided any positive amplification products for the three biosynthetic genes. The three antimicrobial negative isolates from Zijuan still provided positive amplification products *PKS-I* or *PKS-II*.

### Immunomodulatory Activity in Selected Actinobacteria

**FIGURE 3:**
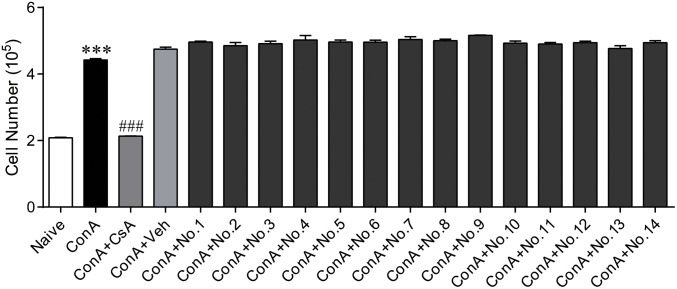
Splenocyte proliferation in ConA-induced splenocytes treated with extracts of endophytic actinobacteria from Zijuan and Yunkang-10. Naive mouse splenocytes were stimulated with 5 μg/mL of ConA in the presence of different extracts, and 500 ng/mL CsA was set as a positive control. No.1-YFT113, No.2-YXN120, No.3-YXN111, No.4-YXN112, No.5-YXT131, No.6-YKFG1221, No.7-YKFG1121, No.8-YKFG1122, No.9-YKFT1130, No.10-YKFH1122, No.11-YKFG1112, No.12-ZFY142, No.13-ZFG130, 14-ZJFT1121. Data are means ± SD (*n* = 5), ^∗∗∗^*p* < 0.001 vs. naive, ^###^*p* < 0.001 vs. ConA.

**FIGURE 4:**
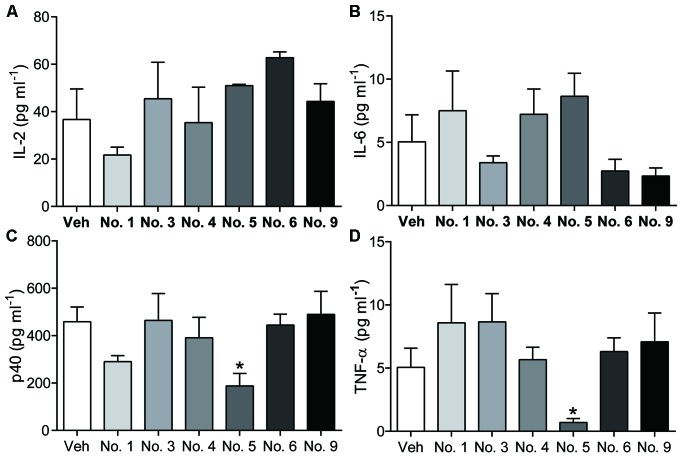
Cytokine levels in sera of mice treated by extracts of endophytic actinobacteria. IL-2 **(A)**, IL-6 **(B)**, IL-12/IL-23 p40 **(C)**, and TNF-α **(D)** levels in serum were measured by ELISA. No.1-YFT113, No.3-YXN111, No.4-YXN112, No.5-YXT131, No.6-YKFG1221, No.9-YKFT1130. Data are means ± SD (*n* = 5), ^∗^*p* < 0.05 vs. vehicle.

Chattopadhyay et al. (2012) showed that black tea has potential anti-inflammatory and immunomodulatory effects in animal models and in human peripheral mononuclear cells, and actinobacteria can produce secondary metabolites with immunosuppressive activity by suppressing cytokine expression and T cell proliferation (Yang et al., 2016). In *in vitro* splenocyte proliferation assays, extracts from 14 actinobacteria that were tested for antibiotic biosynthetic genes showed no inhibition of ConA-induced murine splenocyte proliferation (**Figure 3**), indicating that extracts from actinobacteria might not directly affect splenocyte proliferation. In the animal model, six isolates, YFT113, YXN111, YXN112, YXT131, YKFG1221, and YKFT1130, were examined for their potential immunomodulatory activity. Compared with the control group, BALB/c mice treated by fermentation extracts of the six isolates showed no obvious clinical signs of poisoning or other atypical signs throughout the trial. No significant differences in body weight were observed between control and fermentation extract-treated mice during the 2 weeks of testing (data not shown). CD4^+^ T cells are the key components of the adaptive immune system, and naïve CD4^+^ T cells can differentiate into effector T helper cell subsets (e.g., Th1, Th2, or Th17) by the coordinated functioning of distinct cytokines, including IL-6, IL-12, IL-23, and IL-2 (Murphy and Stockinger, 2010). TNF-α is a multifunctional cytokine that coordinates tissue homeostasis by regulating cytokine production, cell survival, and cell death (Annibaldi and Meier, 2018). In this study, no significant differences in IL-2 and IL-6 concentrations in serum were observed between control and fermentation extract-treated mice (**Figures 4A,B**). IL-12 and IL-23 are heterodimeric cytokines that share a common p40 subunit. To our surprise, the serum levels of IL-12/IL-23 p40 and TNF-α in *Brevibacterium* sp. YXT131 fermentation extract-treated mice were significantly lower than in the control group, but other fermentation extract-treated groups showed no significant differences (**Figures 4C,D**). These results indicated that the isolate YXT131 appeared to have immunosuppressive activity.

## Discussion

As described by previous studies, the natural characteristics and planting environment of Zijuan and Yunkang-10 are similar, and the two cultivars both originate from the Yunnan province of China (Wang et al., 2017; Zhu et al., 2017). The high anthocyanin content of Zijuan is one of the most important differences between the two cultivars (Yang X.R. et al., 2009). Previous studies have indicated that plants secondary metabolites such as alkaloids, phenolics, and terpenoids can interfere with cancer cells, bacteria, and fungi (Wink et al., 2012), and that anthocyanins act as antimicrobial agents of natural plant origin (Cisowska et al., 2011). The different anthocyanin content of Zijuan and Yunkang-10 may influence the microbial community, diversity and bioactivity of endophytic actinobacteria. In this study, the endophytic actinobacteria from Zijuan and Yunkang-10 were isolated and compared for their diversity and antimicrobial and immunomodulatory activities.

In our study, 44 isolates were isolated in July and December, but none of the endophytic actinobacteria was obtained in September. This result might be an incidental, alternatively, endophytic actinobacteria community in Zijuan and Yunkang-10 changed in September and is unculturable on these media. Many studies indicated that the endophytes of plant tissues [such as maple tree sap (Filteau et al., 2010), the buds of Scots pine trees (Pirttilä et al., 2005) and the grape endosphere (Baldan et al., 2014; Bulgari et al., 2014)] were shown to be sensitive to seasonal changes. Other than culture-dependent method, a variety culture independent methods including T-RFLP, PCR fingerprinting and 16S rRNA specific probes were used to investigate the seasonal community changes, and these results were consistent to the present culture-dependent investigation from tea plant (Shen and Fulthorpe, 2015).

In recent years, the antibiotics abuse has been resulted serious bacterial resistance, and become a heavy threat to public health. For instance, methicillin-resistant staphylococcal infections are an important cause of catheter-associated disease, and 75–90% among hospital isolates are *S. epidermidis* (Otto, 2009). Antibiotic resistance by *Shigella* species is also a global issue now (Lampl, 2015). *E. coli* and *B. cereus* are abundant in nature, and many factors make them a potential threat for the food industry (Gundogan and Avci, 2014). Endophytic actinobacteria are well-known producers of a vast array of secondary metabolites, including antibiotics. In our study, all of 44 isolates were screened for antimicrobial activities against *S. epidermidis*, *Sh. flexneri*, *E. coli*, and *B. cereus*. Ten isolates from Yunkang-10 exhibited antimicrobial activity against at least one of the tested pathogenic microorganisms, but none of the isolates from Zijuan showed obvious activity. The high inhibitory activity and broad antimicrobial spectrum of these tested strains suggested that the endophytic actinobacteria from tea cultivar Yunkang-10 are potential candidates for novel antimicrobial agents. For *PKS-I*, *PKS-II*, and *NRPS* screening, the antimicrobial activity results and the biosynthetic genes seemed to be positively correlated in isolates from Yunkang-10, but 3 isolates from Zijuan that showed no antimicrobial activity still provided positive amplification products for *PKS-I* or *PKS-II*. The 3 isolates from Zijuan that showed negative antimicrobial results in this study might produce the antimicrobial agents to other pathogenic microorganisms. The 6 isolates with a broad spectrum of antimicrobial activity and high inhibitory effects were then selected for potential immunomodulatory activities in *in vivo* test. The isolate *Brevibacterium* sp. YXT131, which have broad spectrum antimicrobial activity, gave positive amplification products with *PKS-I*, *PKS-II*, and *NRPS* primers, also exhibited high inhibitory effects of the serum levels of IL-12/IL-23 p40 and TNF-α. These results indicated that endophytic actinobacteria from Yunkang-10 might be an undeveloped bioresource library for active compounds.

## Conclusion

In this study, we found that the endophytic actinobacterial communities in the tea cultivars Zijuan and Yunkang-10 were quite different. The isolates of *Leucobacter* sp. and *Streptomyces* sp. were endemic actinobacterial groups for the Zijuan cultivar, while *Mycobacterium* sp., *Pseudarthrobacter* sp., and *Saccharomonospora* sp. were endemic actinobacterial groups for Yunkang-10. Ten of the 28 isolates (35.7%) from Yunkang-10 exhibited activity against at least one of the tested pathogenic microorganisms, but none of the 16 isolates from Zijuan showed obvious antimicrobial activity. *Brevibacterium* sp. YXT131 and *Brevibacterium* sp. YKFG1221 from Yunkang-10 appeared to have broad-spectrum antimicrobial activity (against *S. epidermidis*, *Sh. flexneri*, and *E. coli*) and gave positive amplification products for the *PKS-I*, *PKS-II*, and *NRPS* genes. The crude extract from *Brevibacterium* sp. YXT131 showed no inhibition of ConA-induced splenocyte proliferation but decreased IL-12/IL-23 p40 and TNF-α levels in the serum of a mouse model, indicating that *Brevibacterium* sp. YXT131 had immunosuppressive activity. Endophytic actinobacteria from Yunkang-10 might be an undeveloped bioresource library for active compounds.

## Author Contributions

JX, XW, and YZ conceived of and designed the experiments. WW, XW, FC, XY, and YL performed the experiments. WW drafted the manuscript. WW, YZ, JX, CW, and XC analyzed the data. JX and XW revised the manuscript. All authors read and approved the final manuscript.

## Conflict of Interest Statement

The authors declare that the research was conducted in the absence of any commercial or financial relationships that could be construed as a potential conflict of interest.

## Footnotes

1 https://blast.ncbi.nlm.nih.gov/Blast.cgi

2 https://www.ezbiocloud.net/

## Supplementary Material

FIGURE S1 DNA gel electrophoresis of *PKS-I*, *PKS-II*, and *NRPS*. The PCR amplification products were resolved using electrophoresis in 1.5% agarose gels. M-Marker, 1-YFT113, 2-YXN120, 3-YXN111, 4-YXN112, 5-YXT131, 6-YKFG1221, 7-YKFG1121, 8-YKFG1122, 9-YKFT1130, 10-YKFH1122, 11-YKFG1112, 12-ZFY142, 13-ZFG130, 14-ZJFT1121.
