# Supplementary figures and images for: Isolation, Diversity, and Antimicrobial and Immunomodulatory Activities of Endophytic Actinobacteria From Tea Cultivars Zijuan and Yunkang-10 (Camellia sinensis var. assamica)
Source: Front Microbiol. 2018 Jun 18;9:1304. doi: 10.3389/fmicb.2018.01304 (PMC6015896; doi:10.3389/fmicb.2018.01304)

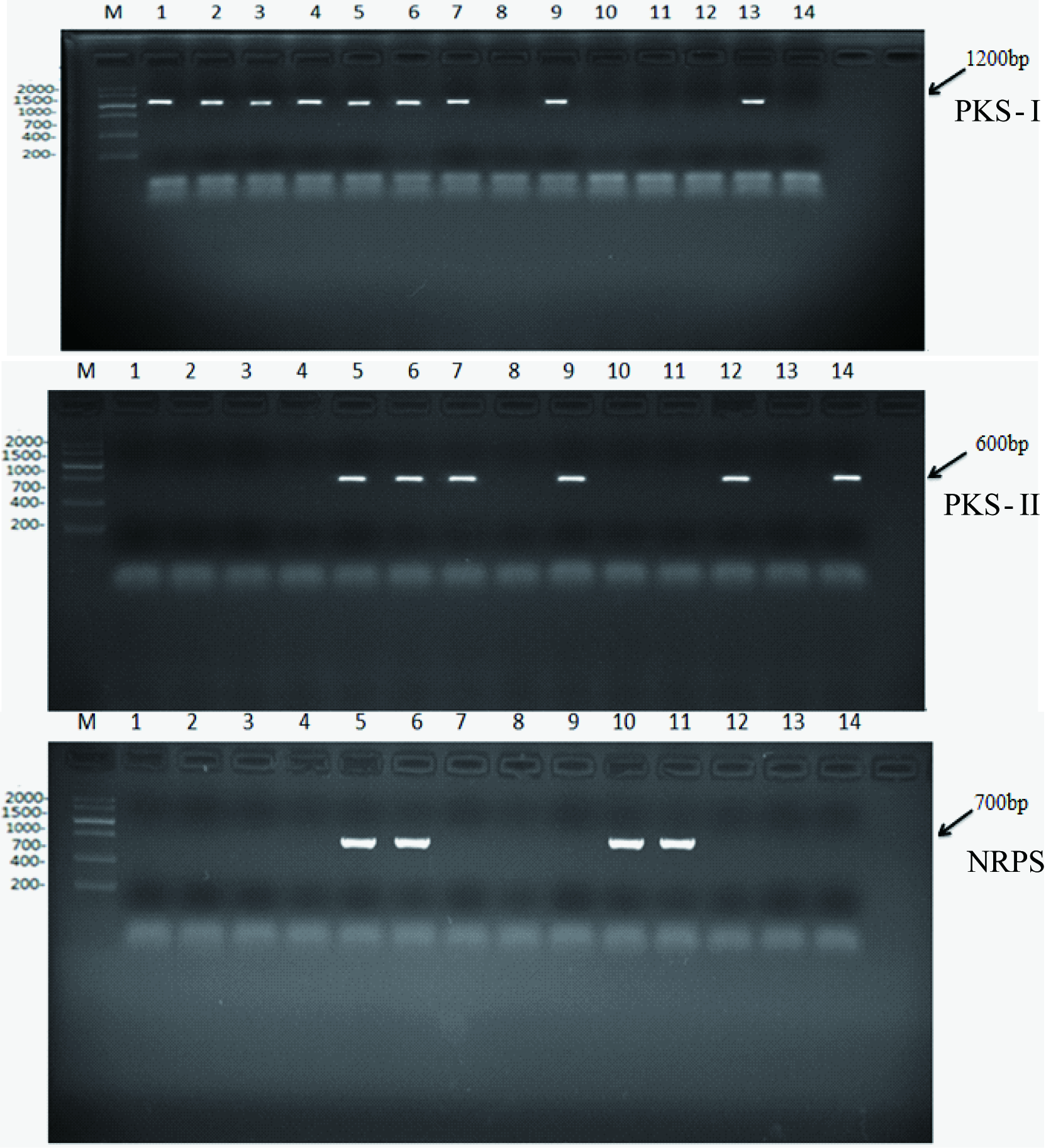

Supplement: FIGURE S1 — DNA gel electrophoresis of PKS-I, PKS-II, and NRPS. The PCR amplification products were resolved using electrophoresis in 1.5% agarose gels. M-Marker, 1-YFT113, 2-YXN120, 3-YXN111, 4-YXN112, 5-YXT131, 6-YKFG1221, 7-YKFG1121, 8-YKFG1122, 9-YKFT1130, 10-YKFH1122, 11-YKFG1112, 12-ZFY142, 13-ZFG130, 14-ZJFT1121. [file Image_1.tif]
